# Giant Cell Arteritis in a 12-Year-Old Girl Presenting with Nephrotic Syndrome

**DOI:** 10.1155/2014/491937

**Published:** 2014-10-21

**Authors:** Zeinab A. El-Sayed, Hanaa M. El-Awady, Zeinab E. Hassan, Tamer M. H. Adham, Hossam M. Mostafa, Nadia G. Elhefnawy

**Affiliations:** ^1^Pediatric Department, Faculty of Medicine, Ain Shams University, Abbassia, Cairo 11566, Egypt; ^2^Pathology Department, Faculty of Medicine, Ain Shams University, Abbassia, Cairo 11566, Egypt

## Abstract

Giant cell arteritis (GCA) is rare in children. The kidneys are generally spared. We present a case of GCA in a 12-year-old girl with severe headache and tender scalp especially over the right temporal area. The right superficial temporal artery was cord like and nodular and the pulsations were barely felt. Several small tender nodular swellings were felt in the occipital area. She had been previously diagnosed as a case of nephrotic syndrome due to underlying membranoproliferative glomerulonephritis. This report is aimed at drawing attention to this rare form of vasculitis in children aiming at decreasing its morbidities.

## 1. Introduction 

Temporal arteritis, also known as giant cell arteritis (GCA), is a systemic, inflammatory vascular syndrome predominantly affecting the cranial arteries. It is rare in children [1]. Eye complications occur at about 3 months and include partial or total blindness, amaurosis fugax, and, on occasion, sudden extraocular muscle dysfunction [2, 3]. The lungs, the abdominal viscera, and the skin may also be involved [3–5]. GCA generally spares the kidneys. However, renal involvement does occur, usually presenting as renovascular hypertension [3]. The diagnosis is a clinical one confirmed with a temporal artery biopsy. In 10–42% of patients eventually diagnosed with GCA, the arteries, by biopsy, do not show signs of inflammation [2, 4], the so-called biopsy negative GCA [6, 7].

## 2. Case Report

A 12-year-old girl presented to the Outpatient Clinic of Children's Hospital, Ain Shams University, Egypt, complaining of severe headache and tenderness over the scalp and right temporal area extending to the occipital region, of gradual onset, progressive course, and a 3-month duration. The headache was bursting, having its summit after school days, and was not associated with vomiting or blurring of vision. Ever since birth, the child was normal. At the age of 9, she suffered recurrent attacks of generalized edema diagnosed at another hospital as nephrotic syndrome for which she received several courses of daily oral prednisone at doses reaching 1.5 mg/kg. The response was incomplete but the patient's mother admitted that her daughter's compliance to therapy was poor and was off therapy for the preceding 2 months.

The child experienced frequent attacks of vertigo, fatigability, and bilateral knee arthralgia. There were no claudications, no Raynaud's phenomenon, and no history of rashes or photosensitivity or other system involvement. There was no history of a preceding viral infection or drug intake, and the patient denied any previous contact with animals. The family history was irrelevant.

**Figure 1:**
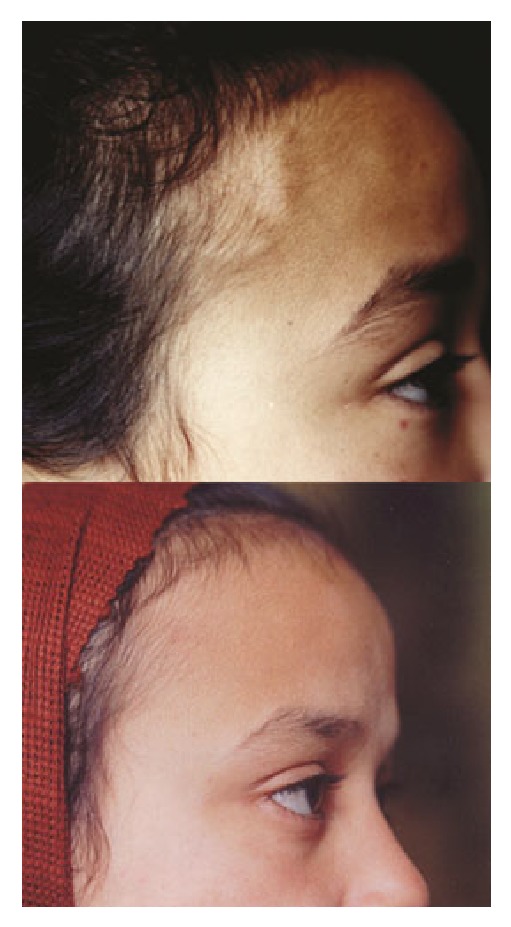
Above: patient's photograph before treatment. The right superficial temporal artery shows dilatation along its course, nodular in some parts. The red papules seen on the forehead and below her right eye are due to insect (mosquito) bites. Below: patient's photograph after treatment. It shows complete disappearance of the right superficial temporal artery abnormalities.

On examination, her general condition was fair; she was fully conscious with puffy eyelids. Her temperature and respiratory rate were normal, but her radial pulse was bounding regularly at a rate of 86/minute and was equal on both sides. Her BP, measured at the arms, was 140/100 mmHg. Her weight was 30 kg and her height was 130 cm. The patient had a cord like nodular right superficial temporal artery over which the pulsations were barely felt and significantly decreased compared to the left side (Figure 1). Several small tender nodular swellings were felt over the occipital area which the mother reported to have noticed a few months before. An audible bruit was heard by auscultation over the artery. The overlying scalp was tender. Pulsations over large arteries were normal with no audible bruits. There were no rashes. Ophthalmologic examination was normal with no audible bruit over the eye balls. There was bilateral pitting lower limb edema up to the thigh and ascites demonstrated by positive shifting dullness. No lipodystrophy was seen. Chest and heart were free. The joints were normal, there was no muscle tenderness, and she was neurologically free.

The investigations revealed high ESR (1st hr, 60 mm), hemoglobin: 10.2 g/dL, total leukocytic count: 6.900/mm^3^, and platelet count: 421.000/mm^3^. Her total serum proteins and albumin were 4.5 gm/dL and 1.4 gm/dL, respectively with a 24-hour proteinuria 2.7 gm and urinary protein/creatinine 4.3. Plasma protein electrophoresis showed hypoalbuminemia with increased alpha_2_ globulin and decreased gamma globulin. Serum creatinine was 0.6 mg/dL and creatinine clearance was 116 mL/minute. There were elevations in serum cholesterol, triglycerides ALT, and AST. The CRP was elevated (24 mg/L), p-ANCA was positive, and complement 3 decreased (41 mg/dL, normal: 55–120 mg/dL). Antinuclear antibodies and anti-DNA were negative. Plain X-ray chest and abdominal ultrasonography were normal.

**Figure 2:**
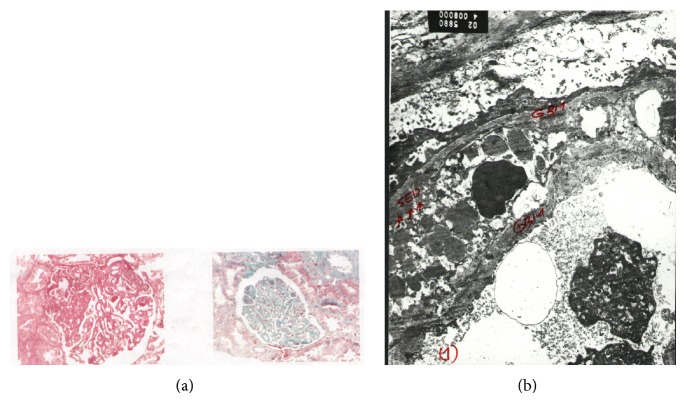
Renal histopathology. (a) Light microscopy. The tufts show marked diffuse thickening of the capillary basement membrane, sometimes amounting to wire-loop configuration. One tuft shows ischemic collapse and another shows a segmental area of proliferation with some nuclear dust. Tubules show focal foam cell transformation. H&E 400x (left) & Masson trichrome 400x (right). (b) Electron microscopy. There are electron dense deposits in the glomerular basement membrane (GBM) in the subendothelial region. Few mesangial and intramembranous deposits are seen as well, 8000x.

An ultrasound-guided renal biopsy showed the following light microscopic findings. Renal tissue examined included 15 glomeruli. Four showed segmental sclerosis and periglomerular fibrosis. Other glomeruli were hypercellular with moderate to severe capillary wall thickening up to wire-loop formation. Foci of tuft necrosis were seen in 3 glomeruli. Hyaline thrombi were frequently seen in capillary lumina and occasional leucocytes. Tubules showed hyaline, RBC, and granular casts. Interstitial tissues showed clusters of foam cells and focal collections of chronic inflammatory cells yet there were no granulomas. Arterioles showed mild to moderate wall thickening. Electron microscopic examination revealed extensive electron dense deposits in subendothelial regions. Few mesangial and intramembranous deposits were seen as well. These findings were consistent with membranoproliferative glomerulonephritis (GN), Figures 2(a) and 2(b).

**Figure 3:**
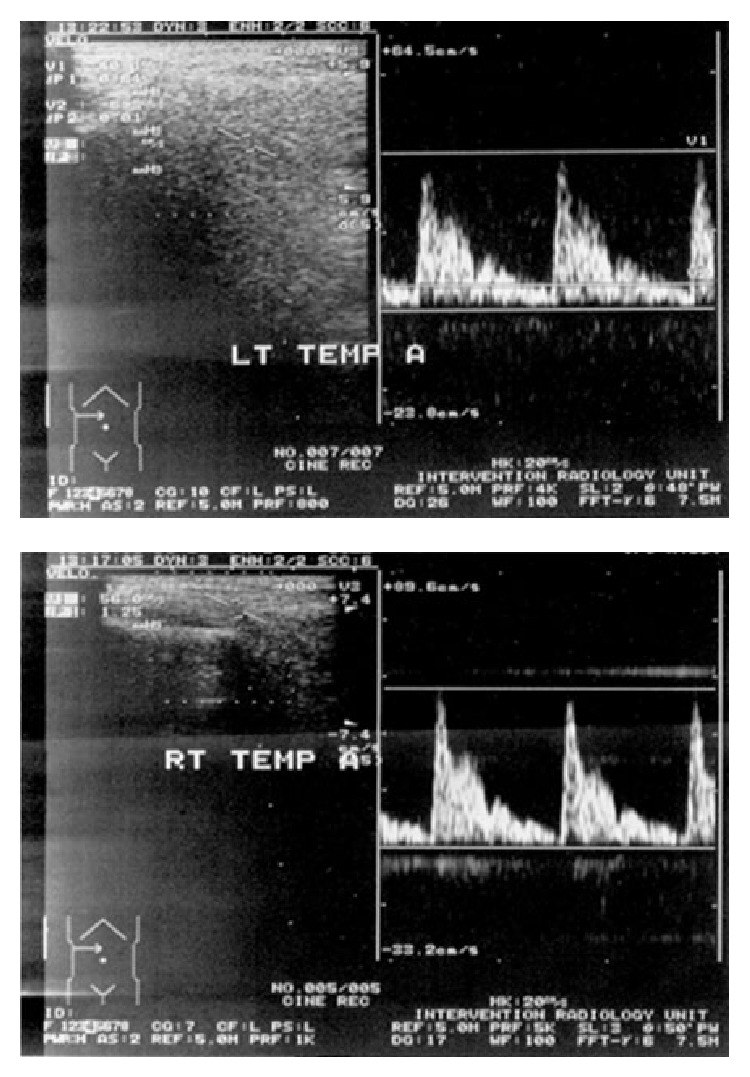
Duplex ultrasound of the temporal arteries. The diameter of the right temporal artery (RT TEMP A) was narrowed compared to the left (LT TEMP A). The resistive index was equal on both.

The hypertension was controlled with antihypertensive drugs, but the headache and superficial temporal artery prominence and nodularity persisted. Hence, a temporal artery biopsy was planned but the parents did not consent to it. Duplex ultrasonography, performed 9 days after institution of steroid therapy, showed the diameter of the right superficial temporal artery to be narrower compared to the left (1.8 mm versus 2 mm) but with the same resistive index to blood flow (Figure 3), whereas the carotid, vertebral, and abdominal arteries were normal.

Treatment was instituted with oral prednisolone at 60 mg/day and the headache and temporal artery abnormalities gradually improved (Figure 1); however, the nephrosis remained unresponsive. Oral cyclophosphamide was started at a dose of 50 mg/day with tapering of steroids and a significant response was observed.

## 3. Discussion 

Giant cell arteritis (GCA) is remarkably rare before the age of 50 years [8]. In the late 19th century, Sir Jonathan Hutchinson reported a man who had difficulty wearing a hat because of tender temporal arteries. Since then, the GCA clinical spectrum has been growing [9]. According to the American College of Rheumatology, the diagnosis of GCA included any three of the following criteria (specificity: 91%): new onset of localized headache, temporal artery tenderness or decreased temporal artery pulse, elevated ESR, arterial biopsy findings of necrotizing arteritis with predominance of mononuclear cell infiltrates or a granulomatous process with multinucleated giant cells, and age greater than or equal to 50 years at disease onset [10]. Other than the age criterion and biopsy findings, the case presented here satisfies the minimum three criteria needed for classification as GCA. GCA was first described in a child in 1977 [11] and there is a report of brachial artery aneurysm in an 8-year-old girl caused by GCA [12]. GCA was described in a 19-year-old woman with vertebral artery aneurysm [13].

The laboratory findings in our patient, though nonspecific, are consistent with those encountered in GCA: elevation of ESR, mild normocytic anemia, and mild thrombocytosis. Thrombocytosis constitutes a risk factor for permanent visual loss in GCA [14]. Additional findings in GCA include increased alpha 2-globulin and transaminases [2]; both were present in our patient.

Temporal artery biopsy has been accepted as the gold standard for diagnosing GCA, though it is of low sensitivity and specificity. Although a minor operation, some patients, as ours, do not agree to it [6]. Current medical practice recommends commencing high dose steroids before performing a biopsy and its continued use even with a negative biopsy, if clinical suspicion is high [15]. Accordingly, therapy was promptly instituted for our patient in spite of the lack of biopsy proven diagnosis.

Color-doppler sonography is a noninvasive tool with high diagnostic accuracy for GCA [16]. The periluminal hypoechoic halo is assumed to represent infiltration and edema in the arterial wall. It might be possible to avoid surgical biopsy in patients with typical sonographic findings [17]. The duplex ultrasound finding of narrow internal diameter of the right superficial temporal artery of our patient is indicative of inflammatory edema of the vessel wall manifesting clinically as dilatation, tortuosity, tenderness, and bruit.

GCA spares the kidneys generally [3]. Rarely, it injures the kidneys, usually by ischemia secondary to vasculitic involvement of the renal arteries or abdominal aorta. This ischemia can cause renovascular hypertension [18]. Glomerulonephritis (GN), whether necrotizing, granular, or membranous, rarely occurs in GCA [5]. Our patient presented first with nephrotic syndrome and neither her clinical nor her laboratory data fitted with SLE. The underlying pathology causing nephrosis in our patient was membranoproliferative GN most probably secondary to GCA. Nephrotic syndrome has rarely been reported in GCA and typically results from secondary renal amyloidosis [19]. Lenz et al. [20] reported a 59-year-old white woman with temporal arteritis who developed progressive renal failure due to focal and segmental necrotizing GN.

Not all arteritis of the temporal arteries are due to GCA. Although rare, juvenile temporal arteritis, in which no systemic disease is noted, may be confused with classic GCA. Physicians should be aware of its benign nature to avoid unnecessary treatment [21]. Bert et al. [22] reported a 9-year-old girl with polyarteritis nodosa presenting initially as temporal arteritis.

In conclusion, GCA does occur in children, albeit rarely. It might present as nephrotic syndrome. Awareness of pediatricians of such rare conditions will help decrease morbidity and mortality among affected children.
